# Supplementary material for: The Cl--channel TMEM16A is involved in the generation of cochlear Ca2+ waves and promotes the refinement of auditory brainstem networks in mice
Source: eLife. 2022 Feb 7;11:e72251. doi: 10.7554/eLife.72251 (PMC8871368; doi:10.7554/eLife.72251)
Supplement: Supplementary file 1. — (a) Comparison of the distribution of interspike intervals (ISIs) between wildtype and cKO mice. (b) Quantification of auditory brainstem response (ABR) thresholds (mean ± SEM) in response to stimulation with tone bursts at 6, 12, and 24 kHz, or click stimulation. (c) Quantification of peak amplitudes (mean ± SEM) of the first three ABR waves (I–III) in response to click stimuli of various intensities (40–100 dB). (d) Quantification of latencies (mean ± SEM) of the first three ABR waves (I–III) in response to click stimuli of various intensities (40–100 dB). [file elife-72251-supp1.docx]

**Supplementary File 1**

**Supplementary file 1a** Comparison of the distribution of ISIs between wildtype and cKO mice.

| **ISI** | **mean No ISIs (%) WT** | **mean No ISIs (%) cKO** | **p-value**  **Chi Square test** |
| --- | --- | --- | --- |
| **0 - 1.0** | 0.00 | 0.08 | 0.3340 |
| **1.0 - 1.58** | 0.09 | 0.00 | 0.3006 |
| **1.58 - 3.98** | 12.05 | 12.45 | 0.7785 |
| **3.98 - 6.31** | 14.72 | 13.17 | 0.3105 |
| **6.31 - 10** | 9.12 | 7.47 | 0.1589 |
| **10 - 15.85** | 6.88 | 2.41 | 2.8664E-07 |
| **15.85 - 25.12** | 6.28 | 2.65 | 2.2086E-05 |
| **25.12 - 39.81** | 7.23 | 2.97 | 1.0653E-07 |
| **39.81 - 63.1** | 6.80 | 4.82 | 0.1347 |
| **63.1 - 100** | 7.92 | 7.63 | 0.8008 |
| **100 - 158.5** | 10.41 | 11.49 | 0.4271 |
| **158.5 - 251.2** | 6.37 | 11.49 | 2.9338E-05 |
| **251.2 - 398.1** | 3.87 | 8.51 | 5.5438E-06 |
| **398.1 - 631** | 3.18 | 4.82 | 0.0458 |
| **631 - 1000** | 2.24 | 2.89 | 0.3178 |
| **1000 - 1585** | 0.95 | 2.17 | 0.0287 |
| **1585 - 2512** | 0.77 | 1.93 | 8.4155E-03 |
| **2512 - 3981** | 0.69 | 0.72 | 0.9200 |
| **3981 - 6310** | 0.26 | 1.12 | 0.0115 |
| **6310 - 10000** | 0.17 | 1.20 | 2.5904E-03 |

**Supplementary file 1b** Quantification of ABR thresholds (mean ± SEM) in response to stimulation with tone-bursts at 6 kHz, 12 kHz, and 24 kHz, or click stimulation.

| **tone frequency (kHz)** | **mean ABR threshold (dB SPL) WT** | **mean ABR threshold (dB SPL) cKO** | **p-value** |
| --- | --- | --- | --- |
| **6 kHz** | 60.0 ± 5.7 | 63.3 ± 3.4 | 0.53, 2way ANOVA |
| **12 kHz** | 43.3 ± 3.7 | 41.4 ± 2.8 |  |
| **24 kHz** | 49.2 ± 3.0 | 54.3 ± 5.3 |  |
| **click** | 45.0 ± 2.5 | 46.4 ± 3.0 | 0.91 (Mann-Whitney) |

**Supplementary file 1c** Quantification of peak amplitudes (mean ± SEM) of the first three ABR waves (I-III) in response to click stimuli of various intensities (40–100 dB).

| **wave** | **click intensity (dB SPL)** | **mean amplitude WT** | **mean amplitude cKO** | **p-value, 2way ANOVA** |
| --- | --- | --- | --- | --- |
| **I** | **50** | 0.78 ± 0.16 | 0.68 ± 0.15 | 0.5175 |
|  | **60** | 1.71 ± 0.30 | 1.47 ± 0.24 |  |
|  | **70** | 2.65 ± 0.38 | 2.59 ± 0.42 |  |
|  | **80** | 3.80 ± 0.60 | 3.56 ± 0.52 |  |
|  | **90** | 4.49 ± 0.75 | 4.17 ± 0.61 |  |
|  | **100** | 4.77 ± 0.80 | 4.59 ± 0.63 |  |
| **II** | **50** | 0.77 ± 0.24 | 0.76 ± 0.16 | 0.3879 |
|  | **60** | 1.30 ± 0.29 | 1.16 ± 0.18 |  |
|  | **70** | 1.24 ± 0.28 | 1.30 ± 0.17 |  |
|  | **80** | 1.66 ± 0.49 | 1.12 ± 0.16 |  |
|  | **90** | 1.43 ± 0.44 | 0.92 ± 0.29 |  |
|  | **100** | 1.37 ± 0.43 | 1.04 ± 0.23 |  |
| **III** | **50** | 0.37 ± 0.04 | 0.31 ± 0.09 | 0.0807 |
|  | **60** | 0.64 ± 0.07 | 0.45 ± 0.13 |  |
|  | **70** | 1.02 ± 0.11 | 0.77 ± 0.19 |  |
|  | **80** | 1.36 ± 0.18 | 0.89 ± 0.21 |  |
|  | **90** | 1.46 ± 0.27 | 1.09 ± 0.25 |  |
|  | **100** | 1.71 ± 0.22 | 1.27 ± 0.27 |  |

**Supplementary file 1d** Quantification of latencies (mean ± SEM) of the first three ABR waves (I – III) in response to click stimuli of various intensities (40–100 dB).

| **wave** | **click intensity (dB SPL)** | **mean latency (ms)**  **WT** | **mean latency (ms)**  **cKO** | **p-value**  **2way ANOVA** |
| --- | --- | --- | --- | --- |
| **I** | **50** | 2.02 ± 0.03 | 2.02 ± 0.03 | 0.9073 |
|  | **60** | 1.83 ± 0.03 | 1.83 ± 0.03 |  |
|  | **70** | 1.71 ± 0.04 | 1.71 ± 0.04 |  |
|  | **80** | 1.64 ± 0.04 | 1.64 ± 0.04 |  |
|  | **90** | 1.55 ± 0.03 | 1.55 ± 0.03 |  |
|  | **100** | 1.47 ± 0.03 | 1.47 ± 0.03 |  |
| **II** | **50** | 3.26 ± 0.067 | 3.30 ± 0.11 | 0.1225 |
|  | **60** | 3.05 ± 0.07 | 3.21 ± 0.11 |  |
|  | **70** | 2.02 ± 0.07 | 2.99 ± 0.11 |  |
|  | **80** | 2.83 ± 0.09 | 2.91 ± 0.12 |  |
|  | **90** | 2.75 ± 0.06 | 2.83 ± 0.16 |  |
|  | **100** | 2.71 ± 0.08 | 2.89 ± 0.17 |  |
| **III** | **50** | 4.56 ± 0.11 | 4.74 ± 0.19 | 0.9147 |
|  | **60** | 4.42 ± .012 | 4.40 ± 0.14 |  |
|  | **70** | 4.24 ± 0.09 | 4.20 ± 0.09 |  |
|  | **80** | 4.17 ± 0.12 | 4.08 ± 0.09 |  |
|  | **90** | 4.01 ± 0.07 | 4.06 ± 0.05 |  |
|  | **100** | 3.94 ± 0.08 | 3.90 ± 0.06 |  |
