# Supplementary material for: The Cl--channel TMEM16A is involved in the generation of cochlear Ca2+ waves and promotes the refinement of auditory brainstem networks in mice
Source: eLife. 2022 Feb 7;11:e72251. doi: 10.7554/eLife.72251 (PMC8871368; doi:10.7554/eLife.72251)
Supplement: Transparent reporting form [file elife-72251-transrepform1.pdf]

Westbrook Centre
Milton Road
Cambridge CB4 1YG, UK
P 01223 855340
W elifesciences.org
T @elife

# *eLife's* transparent reporting form

We encourage authors to provide detailed information *within their submission* to facilitate the interpretation and replication of experiments. Authors can upload supporting documentation to indicate the use of appropriate reporting guidelines for health-related research (see EQUATOR Network), life science research (see the BioSharing Information Resource), or the ARRIVE guidelines for reporting work involving animal research. Where applicable, authors should refer to any relevant reporting standards documents in this form.

If you have any questions, please consult our Journal Policies and/or contact us: editorial@elifesciences.org.

**Sample-size estimation**

- You should state whether an appropriate sample size was computed when the study was being designed
- You should state the statistical method of sample size computation and any required assumptions
- If no explicit power analysis was used, you should describe how you decided what sample (replicate) size (number) to use

Please outline where this information can be found within the submission (e.g., sections or figure legends), or explain why this information doesn't apply to your submission:

No statistical test was used to predetermine the sample size. This information can be found in the Materials and Methods section (subsection statistics and statistical significance).

**Replicates**

- You should report how often each experiment was performed
- You should include a definition of biological versus technical replication
- The data obtained should be provided and sufficient information should be provided to indicate the number of independent biological and/or technical replicates
- If you encountered any outliers, you should describe how these were handled
- Criteria for exclusion/inclusion of data should be clearly stated
- High-throughput sequence data should be uploaded before submission, with a private link for reviewers provided (these are available from both GEO and ArrayExpress)

Please outline where this information can be found within the submission (e.g., sections or figure legends), or explain why this information doesn't apply to your submission:

We exclusively performed biological replications following the definition in Blainey et al., Nat Methods, 2014 11:879–80: "Biological replications are measurements of biologically distinct samples that show biological variation (technical replicates are considered repeated measurements of the same sample to assess independent measures of the noise associated with the equipment and the protocols)". Information about the replications can be found in the Results section (text and figures) for each experiment, if applicable. All data (including outliers) were included in the analysis.

eLife Sciences Publications, Ltd is a limited liability non-profit non-stock corporation incorporated in the State of Delaware, USA, with company number 5030732, and is registered in the UK with company number FC030576 and branch number BR015634 at the address Westbrook Centre, Milton Road, Cambridge CB4 1YG.

**Statistical reporting**

- Statistical analysis methods should be described and justified
- Raw data should be presented in figures whenever informative to do so (typically when N per group is less than 10)
- For each experiment, you should identify the statistical tests used, exact values of N, definitions of center, methods of multiple test correction, and dispersion and precision measures (e.g., mean, median, SD, SEM, confidence intervals; and, for the major substantive results, a measure of effect size (e.g., Pearson's r, Cohen's d)
- Report exact p-values wherever possible alongside the summary statistics and 95% confidence intervals. These should be reported for all key questions and not only when the p-value is less than 0.05.

Please outline where this information can be found within the submission (e.g., sections or figure legends), or explain why this information doesn't apply to your submission:

> The criteria used for each statistical test, N numbers, the method of multiple test correction, and dispersion measures are stated in the Materials and Methods section (subsection statistics and statistical significance).
> For each dataset, the statistical method used, the value of N, and the p-value is stated in the corresponding text and figure legend of the results section. If the data were normally distributed the results are displayed as means ± standard error of means and otherwise as medians with the respective 25 % and 75 % quartiles. All p-values are reported as exact values.

(For large datasets, or papers with a very large number of statistical tests, you may upload a single table file with tests, Ns, etc., with reference to sections in the manuscript.)

**Group allocation**

- Indicate how samples were allocated into experimental groups (in the case of clinical studies, please specify allocation to treatment method); if randomization was used, please also state if restricted randomization was applied
- Indicate if masking was used during group allocation, data collection and/or data analysis

Please outline where this information can be found within the submission (e.g., sections or figure legends), or explain why this information doesn't apply to your submission:

> Two distinct genotypes were used (wildtype and *Tmem16a* ear conditional knockout mice). Where possible, littermates were used, which is indicated in the respective section of the results part. Grouping of data was performed per genotype.
> For the analysis of MNTB-LSO input maps, the criteria used to define responsive stimulation sites and their grouping is described in the Materials and Methods section (subsection reconstruction and analysis of MNTB-LSO input maps).
> Data analysis was performed by a person blinded to the genotype.

**Additional data files ("source data")**

- We encourage you to upload relevant additional data files, such as numerical data that are represented as a graph in a figure, or as a summary table
- Where provided, these should be in the most useful format, and they can be uploaded as "Source data" files linked to a main figure or table
- Include model definition files including the full list of parameters used

- Include code used for data analysis (e.g., R, MatLab)
- Avoid stating that data files are "available upon request"

Please indicate the figures or tables for which source data files have been provided:

Figure 1
Figure 2
Figure 3
Figure 4
Figure 5
Supplemental Figure 2
Supplemental Figure 5
Supplemental Figure 6

eLife Sciences Publications, Ltd is a limited liability non-profit non-stock corporation incorporated in the State of Delaware, USA, with company number 5030732, and is registered in the UK with company number FC030576 and branch number BR015634 at the address Westbrook Centre, Milton Road, Cambridge CB4 1YG.
